# Are the criteria always right? Assessment of hepatocellular carcinoma cases in living donor liver transplantation at a high-volume center

**DOI:** 10.3906/sag-2101-51

**Published:** 2021-10-21

**Authors:** Yücel YANKOL, Gültekin HOŞ, Turan KANMAZ, Nesimi MECİT, Yılmaz ÇAKALOĞLU, Münci KALAYOĞLU, Koray S. ACARLI

**Affiliations:** 1 Division of Transplantation, Department of Surgery, School of Medicine and Public Health, University of Wisconsin, Madison, WI United States; 2 Organ Transplant Center, Memorial Şişli Hospital, İstanbul Turkey; 3 Department of Surgery, Şişli Etfal Training and Research Hospital, İstanbul Turkey; 4 Organ Transplant Center, School of Medicine, Koç University, İstanbul Turkey

**Keywords:** Hepatocellular carcinoma, living donor liver transplantation, criteria, outcomes

## Abstract

**Background/aim:**

With the increased experience in living donor liver transplantation (LDLT), it has been adopted for the treatment of hepatocellular carcinoma (HCC), with emerging discussions of criteria beyond tumor size and number. In contrast to deceased donor liver transplantation (DDLT), recipient selection for LDLT is not limited by organ allocation systems. We discuss herein the assessment, criteria, and experience with liver transplantation (LT) in HCC cases at a high-volume LDLT center.**Material and methods:** Between August 2006 and December 2017, 191 adult LT HCC recipients with at least one-year follow-up were retrospectively analyzed.

**Results:**

In 191 patients, one-, three- and five-year survival rates were 87.2%, 81.6%, and 76.2%, respectively, including early postoperative mortality. In 174 patients with long-term follow-up, one-, three- and five-year disease-free survival rates were 91.6%, 87.7%, and 84.4%, respectively. When multivariate analysis was utilized, tumor differentiation was the only factor which statistically affected survival (p = 0.025).

**Conclusion:**

LDLT allows us to push the limits forward and the question “Are the criteria always right?” is always on the table. We can conclude that, with the advantage of LDLT, every HCC patient deserves a case-by-case basis discussion for LT under scientific literature support. In borderline cases, tumor biopsy might help determine the decision for LT.

## 1.Introduction 

Hepatocellular carcinoma (HCC) is the most common primary liver cancer and remains an ongoing problem, with incidence increasing worldwide. It is also well known that HCC develops mainly in chronically diseased livers, with low median survival rates if no treatment is received [1–3]. There are various modalities for curative and palliative treatment. Surgical resection and interventional radiological treatment are the options with successful outcomes in limited cases due to underlying chronic liver disease. During the last decades, liver transplantation (LT) became a radical treatment for HCC in that it can simultaneously treat intrahepatic metastasis as well as multicentric carcinogenesis and diseased liver [4–6].

During the last two decades, Milan Criteria (MC) has been implemented worldwide for LT in cases of HCC, and many organ sharing programs now use MC for organ allocation. Starting with the University of California, San Francisco (UCSF) criteria [7], over the past decade, the search for new criteria and discussions of LT algorithms for HCC became a hot topic in the field. With increased experience in living donor transplantation (LDLT), LDLT was adopted in the setting of HCC treatment with new discussions about criteria beyond the size and number of tumors. In contrast to deceased donor liver transplantation (DDLT), recipient selection for LDLT is not limited by organ allocation systems. 

In this study, we discuss the criteria for LT in HCC cases, sharing our experience and assessment of our HCC cases as a high volume LDLT center.

## 2. Material and methods

Between August 2006 and December 2017, 1,067 LTs (890 LDLT, 177 DDLT) were performed in 1027 patients (704 adult, 323 pediatric) at our center. Following Institutional Review Board approval, patient data was collected retrospectively in 208 HCC LT patients. Pediatric patients (<18 years), patients with other liver malignancies combined with HCC, and patients lost to follow-up were excluded. A total of 191 adult LT HCC recipients with a minimum one-year follow-up were retrospectively analyzed. Demographics, underlying liver disease, tumor related radiological (total tumor size, total number of tumors, largest tumor size) and pathological data (macro- and microvascular invasion, tumor differentiation), AFP levels, recurrence and survival rates) were recorded and analyzed. In addition, patients were classified according to MC and USCF criteria. SPSS version 13.0 (SPSS, Inc., Chicago, IL, USA) was used for the Kaplan Meier Survival and Cox Regression Multivariate analysis. 

### 2.1. Patient evaluation and selection

Starting from the beginning of the transplant program, all chronic liver disease patients with HCC were evaluated in the multidisciplinary selection meeting as possible LT candidates. Beyond tumor size and number, patients who did not have findings of extrahepatic or macrovascular invasion, tumor thrombosis, lymphatic node or findings of extrahepatic metastasis were evaluated as LT candidates. During the evaluation and selection process, cases within MC were approved both for DDLT and LDLT. Patients were listed for DDLT according to the Turkish Health Ministry organ allocation system rules and were asked about potential related living liver donors. Cases beyond MC were evaluated for LDLT according to their additional findings. During this evaluation, beyond the tumor size and number, we focused on findings which could give us an idea about the biological behavior of the tumor. Tumor growth rate and time, AFP level, tumor margin findings at computed tomography (CT) or magnetic resonance imaging (MRI) views, positron emission tomography and computed tomography (PET-CT) findings, response to other previous treatments, histopathological differentiation (if there was a biopsy) and age of the patients were the parameters, which we interpreted before making a decision. One or two parameters supporting poor biological behavior were not enough to make a decision against LT. If most of the findings supported poor biological behavior, alternative and bridging therapies (transarterial chemoembolization - TACE, transarterial radioembolization –TARE, and external beam radiation) options were preferred instead of LT. In addition, all the possibilities and risks were discussed at length with the recipient, living donor candidate and family members. Our living donor selection criteria and outcomes were previously published [8]. 

### 2.2.Immunosuppression

The protocol for immunosuppressive therapy was triple maintenance immunosuppressive therapy at the beginning, with a lower dose consisting of prednisone, tacrolimus (Prograf, Astellas USA, Deerfield, IL), and mycophenolate mofetil (Cell-Cept, Roche Laboratories, Nutley, NJ). Prednisolone was stopped in all cases with a taper at one month after transplant, and MMF was stopped in most cases at three months after transplant. Most patients with low tacrolimus levels (4–6 ng/mL) were followed postoperatively according to their clinical findings. In some HCC recurrence cases, mTor inhibitor was started according to the decision made together with the oncologist and hepatologist.

### 2.3.Follow-up after LT 

A thoraco-abdominal CT or/and MRI were performed every 3 months for the first year of follow-up, every 6 months between 1 and 3 years, and annually after 3 years. AFP and clinical examination were performed every month during the first 6 months and every 2 months between 6 months and 1 year of follow-up, every 3 months between 1 and 3 years, and every 6 months between 3 and 5 years. After 5 years, CT or MRI with AFP test was performed annually or if clinically indicated. A biopsy of all suspicious lesions was performed for recurrence, and we attempted to treat all recurrent lesions with surgical resection or interventional radiological treatment after the determination of recurrence.

## 3. Results

Of the 191 cases, the mean age was 56.2 years (18–74 years), and 81.7% (n = 156) of patients were male. Only 14.1% (n = 27) were older than 65 years of age. The main primary liver disease was chronic hepatitis B infection (61.8%), followed by chronic hepatitis C infection (19.4%). Most (66%) LT was performed from a living related liver donor. Mean physiological model for end-stage liver disease (MELD) score was 12.8 (6–29). Mean alpha fetoprotein (AFP) level was 904 ng/mL (1–100,000 ng/mL), but the median AFP level was 10.8ng/mL. According to the preoperative radiological findings and evaluation, 115 (60%) of the 191 pathologically HCC approved cases were within the MC (rMC), 45 (24.6%) were beyond the MC (rMC), and 31 (16.2%) were incidentally discovered HCC (iHCC) cases upon histopathological examination. After histopathological examination, 120 (62.8%) cases within the MC (pMC) and 71 (37.2%) were beyond the MC (pMC). In addition, 54 (28.3%) of the all cases were beyond the USCF criteria (Table 1).

**Table 1:** Demographics.

	Within pMCn = 120	Beyond pMCn = 71	Within USCFn = 137	Beyond USCFn = 54	Totaln = 191
Mean Age (years)	56.5	56.0	56.4	55.9	56.2
Sex ( %, n )					
Female	20.0 % (24)	15.5 % (11)	17.5 % (24)	20.4 % (11)	18.3% (35)
Male	80.0 % (96)	84.5 % (60)	82.5 % (113)	79.6 % (43)	81.7% (156)
Mean MELD Score	13.1	12.1	12.8	12.7	12.8
Mean AFP (ng/mL)	218	2064	197	2697	904
Median AFP (ng/mL)	6	22	5.9	27.4	8
Primary Liver Disease					
HBV & HCV	84.2 % (101)	77.5 % (55)	83.9 % (115)	75.9 % (41)	81.7 % (156)
Others	15.8 % (19)	22.5 % (16)	16.1 % (22)	24.1 % (13)	18.3 % (35)
Early mortality ( %, n)	9.2 % (11)	8.5 % (6)	8.0 % (11)	11.1 % (6)	8.9 % (17)

AFP: alpha-fetoprotein, MELD : the model for end-stage liver disease, HBV: chronic hepatitis B virus, HCV: chronic hepatitis C virus, USCF: University of California, San Francisco criteria, pMC: pathological Milan criteria.

Early postoperative mortality (first 6 months) occurred due to sepsis, primary nonfunction (PNF), multiorgan failure (MOF), cardiac arrest, and neurological complications in 17 (8.9%) cases. These cases were included in the analysis. Of the 17 cases, 9 were within in the pMC and 8 were beyond the pMC (4 of them were beyond the USCF criteria). In addition, 8 were transplanted from a deceased donor, and 9 were transplanted from a living donor.

Of the 191 patients, there were 26 (13.6%) with recurrent disease. Overall mortality was 20,9% (40/191). When early mortalities (n = 17) are excluded, the adjusted long-term mortality dropped to 13.2% (23/174) in HCC recipients with at least one year of follow-up. In 174 patients with long-term follow-up, 1-, 3- and 5-year disease-free survival rates were 91.6%, 87.7%, and 84.4%, respectively. 

With the inclusion of early postoperative mortalities, 1-, 3- and 5-year survival rates were 87.2%, 81.6%, and 76.2%, respectively. In 115 patients within rMC, 1-, 3- and 5-year survival rates were 87.6%, 84.3%, and 79.1%, respectively. In 45 patients beyond the rMC, 1-, 3- and 5-year survival rates were 84.2%, 73.7%, and 63.2%, respectively. In 31 iHCC patients, 1-, 3- and 5-year survival rates were 90.3%, 83.4%, and 83.4%, respectively. In 120 within pMC patients, 1-, 3- and 5-year survival rates were 89.1%, 85.2%, and 80.6%, respectively; and in 71 beyond the pMC patients, 1-, 3- and 5-year survival rates were 84.1%, 76.02%, and 69.4%, respectively. There were no differences between the within versus beyond the rMC (p = 0.18) and within versus beyond pMC (p = 0.12). When data were analyzed according to the pathological UCSF criteria, in 137 within USCF patients, 1-, 3- and 5-year survival rates were 89.5 %, 85.4%, and 81.2%, respectively, and, in 54 beyond the UCSF patients 1-, 3- and 5-year survival rates were 81.3%, 72.5%, and 64.5%, respectively. There were statistical survival differences between within UCSF patients versus beyond the UCSF patients (p = 0.029) (Table 2). 

**Table 2:** Kaplan–Meier survival comparison between subgroups.

	First group (n) - 1, 3 and5 year Survival Rates	Second group (n) 1, 3 and5 year Survival Rates	p Value
Radiological	Within rMC (n = 115)	Beyond rMC (n = 45)	0.18
Milan Criteria(rMC)	1 year 87.6%	1 year 84.2%	
	3 year 84.3%	3 year 73.7%	
	5 year 79.1%	5 year 63.2%	
Pathological	Within pMC (n = 120)	Beyond pMC (n = 71)	0.12
Milan Criteria(pMC)	1 year 89.1%	1 year 84.2%	
	3 year 85.2%	3 year 76.0%	
	5 year 80.6%	5 year 69.4%	
USCF Criteria	Within USCF (n = 137)	Beyond USCF (n = 54)	0.029
	1 year 89.5%	1 year 81.3%	
	3 year 85.4%	3 year 72.5%	
	5 year 81.2%	5 year 64.5%	
AFP Level	AFP < 200 ng/mL (n = 165)	AFP ≥ 200 ng/mL (n = 26)	0.89
	1 year 87.6%	1 year 84.6%	
	3 year 81.7%	3 year 80.8%	
	5 year 76.2%	5 year 75.7%	
Total Tumor Size	tTs < 8 cm (n = 150)	tTs ≥ 8 cm (n = 41)	0.19
(tTs)	1 year 87.8%	1 year 88.7%	
	3 year 83.1%	3 year 83.1%	
	5 year 79.2%	5 year 69.8%	
Recipient Age	Age < 65 ( n = 164 )	Age ≥ 65 (n = 27)	0.016
	1 year 89.5%	1 year 72.0%	
	3 year 84.4%	3 year 64.7%	
	5 year 79.1%	5 year 58.8%	
Microvascular	MVI (-) (n = 120)	MVI (+) (n = 68)	0.004
Invasion (MVI)	1 year 90.8%	1 year 80.4%	
	3 year 86.8%	3 year 71.8%	
	5 year 84.0%	5 year 61.6%	

AFP: alpha-fetoprotein, MELD : the model for end-stage liver disease, USCF: University of California, San Francisco criteria.

When the data were analyzed according to total tumor numbers (1, 2, 3, 4-9 and more than 10 tumors), there was not a significant difference between the five groups (p = 0.54) (Figure 1A). There were 13 cases with long-term follow-up with more than 10 tumors; 3 deaths occurred due to HCC recurrence in a total 6 cases with recurrence (Table 3). We also instituted a cut-off for total tumor size of 8 cm, as this was the most supported limit [9] in the literature, and there were not significant differences between total tumor size over and below 8 cm (p = 0.19) (Table 2). With our evaluation system, we had a chance to transplant only seven patients with the largest tumor size more than 8 cm. Statistically, our case number was not large enough to make a conclusion, but 5 lived for more than 5 years and 3 are still living without HCC recurrence more than 5 years posttransplant (Table 4). We did not find any significant differences in our patient population with AFP levels higher and lower than 200ng/mL (p = 0.89) (Table 2). There were only 16 cases followed long-term with AFP ≥400 ng/mL, and two deaths occurred due to HCC recurrence in a total of 6 cases with recurrence (Table 5). In our HCC patients, MELD scores of the recipients did not affect survival rates by subgroup (p = 0.72). According to our univariate analysis, poor tumor differentiation (p = 0.0001) (Figure 1B), microvascular invasion (p = 0.004)(Table 2) and recipient age ≥65 (p = 0.016) (Table 2) affected patient survival. Comparably with all our LT patients, older HCC (age ≥65) recipient survival rates at 1, 3 and 5 years (72.0%, 64.7% and 58.8%, respectively) were significantly lower than those for younger recipients (age <65) survival rates (89.5%, 84.4% and 79.1%, respectively) (Table 2). When Cox regression multivariate analysis was performed, including all the factors, tumor differentiation was the only factor, which statistically affected survival in our patients (p = 0.025) (Table 6). Although our case number was not large enough to reach statistical significance, largest tumor size greater than 8 cm increased the overall HCC recurrence rate (57.1%, n:4/7) and decreased the long-term overall patient survival rate (71.4%, n:5/7) (Table 4). 

**Table 3:** Transplant patients followed long term with tumor number ≥10.

	Tm Number	Age	TmDiff.	AFP(ng/mL)	Biggest Tm size	Rec	Rec.time (month)	Status	Post LT Year
1	>10	18	M	100000	7.0	Yes	14	Dead	3.1
2	>10	56	W	180	11.5	No	-	Alive	11.8
3	>10	54	M	234	6.0	No	-	Alive	9.6
4	>10	47	M	5	4.5	No	-	Alive	8.5
5	>10	61	M	3	2.8	Yes	11	Dead	2.6
6	>10	68	M	137	8.0	Yes	55	Alive	7.6
7	>10	29	M	341	2.5	Yes	35	Alive	7.2
8	>10	53	W	6	3.0	No	-	Alive	6.7
9	>10	62	M	6	10.0	Yes	36	Dead	3.2
10	>10	65	M	1426	3.5	No	-	Alive	6.7
11	>10	19	W	727	0.2	No	-	Alive	6.8
12	>10	61	M	7175	3.5	Yes	11	Alive	3.3
13	>10	59	M	2	3.7	No	-	Alive	1.8

AFP: alpha-fetoprotein, Tm:tumor, Diff: differentiation, Rec: recurrence, LT: liver transplant, W: well differentiated tumors M; moderately differentiated tumors.

**Table 4:** Transplant patients followed long term with largest tumor ≥7 cm.

	LargestTm size	Age	TmDiff.	AFP(ng/mL)	Tm Number	Rec	Rec.time (month)	Status	Post LT Year
1	11.5	37	M	12520	1	No	-	Alive	9.1
2	10.0	62	W	6	>10	Yes	36	Death	3.2
3	10.0	51	M	1	7	Yes	4	Death	2.5
4	8.7	51	M	7447	1	Yes	9	Alive	5.6
5	8.0	55	M	1779	1	No	-	Alive	8.8
6	8.0	68	M	137	>10	Yes	55	Alive	7.6
7	8.0	57	W	15	1	No	-	Alive	8.8
8	7.2	60	P	9946	3.0	No	-	Alive	3.7
9	7.0	56	W	180	>10	No	-	Alive	11.8
10	7.0	51	M	6	1	Yes	6	Death	1.3
11	7.0	68	M	2	2	No	-	Alive	9.9

AFP: alpha-fetoprotein, Tm:tumor, Diff: differentiation, Rec:recurrence, LT:liver transplant, W: well differentiated tumors, M:moderately differentiated tumors, P:poorly differentiated tumors

**Table 5:** Transplant patients followed long term with AFP level ≥400 mg/mL.

No	AFP(ng/mL)	Age	TmDiff.	Criteria	Tm Number	Largest Tm size	Rec	Rec. time (month)	Status	Post LT Year
1	100000	18	M	B USCF	>10	5.0	Yes	14	Death	3.1
2	12520	37	M	B USCF	1	11.5	No	-	Alive	9.1
3	9946	60	P	B USCF	1	7.2	No	-	Alive	3.7
4	7447	51	M	B USCF	1	8.7	Yes	9	Alive	5.6
5	7325	55	M	MC	1	4.5	No	-	Alive	10.7
6	7175	61	M	B USCF	>10	3.5	Yes	11	Alive	3.3
7	3893	62	M	MC	1	3.5	No	-	Alive	3.1
8	2072	66	P	MC	1	3.3	Yes	4	Death	0.7
9	1799	55	M	B USCF	1	8.0	No	-	Alive	8.8
10	1426	65	M	B USCF	>10	3.5	No	-	Alive	6.7
11	1358	55	M	MC	1	4.0	Yes	72	Alive	12.0
12	1000	64	M	B USCF	2	5.0	No	-	Alive	5.1
13	727	19	W	B USCF	>10	0.2	No	-	Alive	6.8
14	721	65	P	B USCF	4	2.9	No	-	Alive	4.2
15	551	69	M	MC	1	4.5	Yes	13	Alive	2.9
16	497	66	M	USCF	2	3.8	No		Alive	7.9

AFP: alpha-fetoprotein, Tm: tumor, Rec: recurrence, LT: liver transplant, MC: Within Milan criteria, B USCF: Beyond The University of California, San Francisco criteria, USCF: Within The University of California, San Francisco criteria, Diff: differentiation W: well differentiated tumors, M: moderately differentiated tumors, P: poorly differentiated tumors.

**Table 6:** Cox-regression multivariate analysis.

	B	SE	Wald	df	P Value (Sig)	Exp(B)
rMC	0.066	0.372	0.031	1	0.859	1.068
pMC	0.913	0.771	1.403	1	0.236	2.469
USCF	–0.993	0.718	1.911	1	0.167	0.371
AFP Level (200ng/mL)	–0.594	0.485	1.503	1	0.220	0.552
Tm differentiation	0.800	0.357	5.009	1	0.025	2.225
Microvascular invasion	0.461	0.371	1.537	1	0.215	1.585
Tm number	–0.100	0.178	0.318	1	0.573	0.905
Total tm size (8 cm)	0.083	0.530	0.025	1	0.875	1.087
Recipient MELD score	0.163	0.151	1.155	1	0.282	1.177
Recipient age (65)	0.476	0.390	1.489	1	0.222	1.609

AFP: alpha-fetoprotein, MELD: the model for end-stage liver disease, USCF: University of California, San Francisco criteria, Tm: tumor, rMC: radiological Milan criteria, pMC: pathological Milan criteria.

**Figure 1a:**
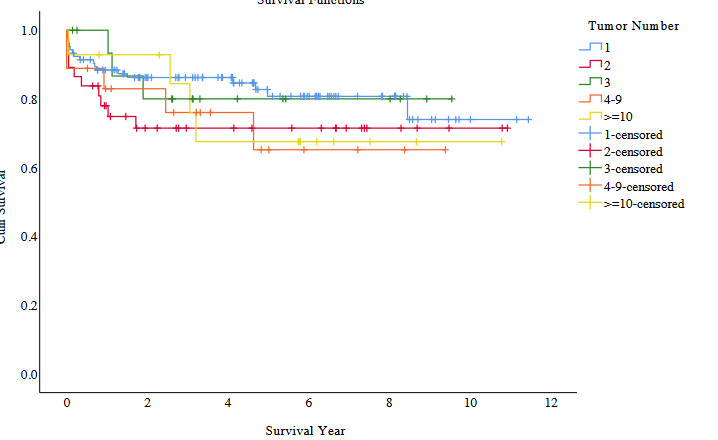
Survival comparison with Kaplan–Meier between the 5 groups of number of tumors (1,2,3,4–9 and more than 10).

**Figure 1b:**
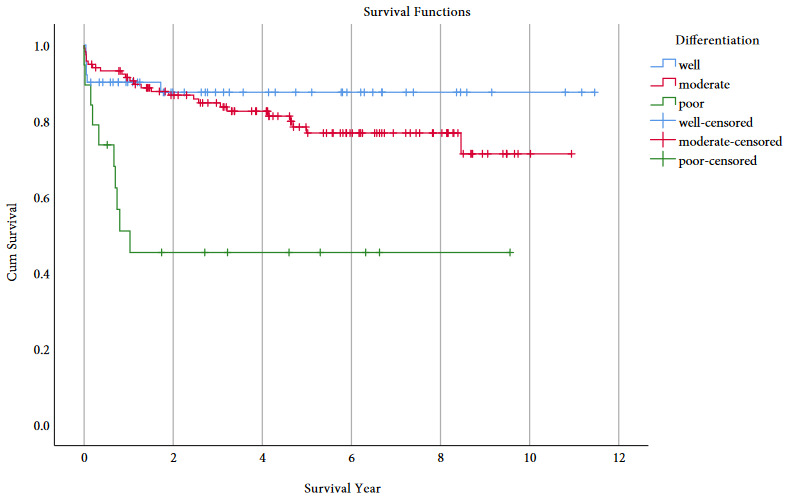
Survival comparison with Kaplan–Meier between the 3 groups of tumor differentiation (well, moderate and poor).

In our HCC patients with recurrence, 1-, 3- and 5-year survival rates were 81.3%, 54.7%, and 25.0%, respectively (Figure 2A). Of the 50 beyond UCSF patients with long-term follow-up for well-differentiated tumors (n = 10), 1-, 3- and 5-year survival rates were all 90% and, for moderately differentiated tumors (n = 32), 1-, 3- and 5-year survival rates were 84.1%, 76.7%, and 67.3%, respectively. In this group, for poorly differentiated tumors (n = 8), survival rates dropped to 46.0% at 1 year and 31.3% at 2 years (Figure 2B). 

**Figure 2a:**
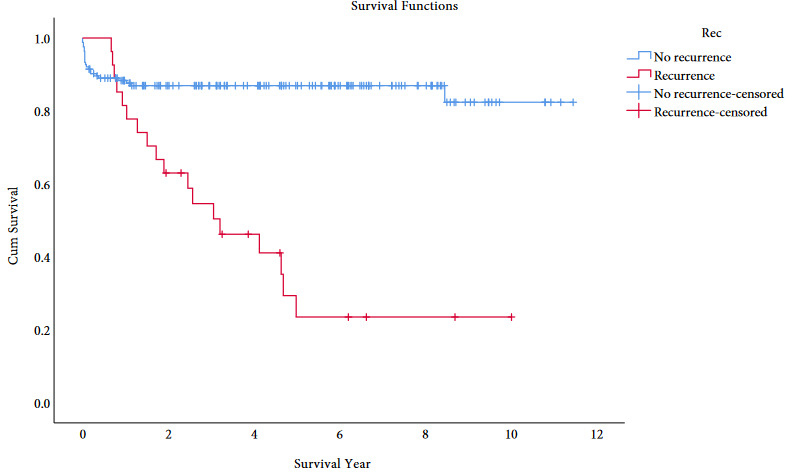
Survival comparison with Kaplan–Meier between the tumor recurrence and nonrecurrence groups.

**Figure 2b:**
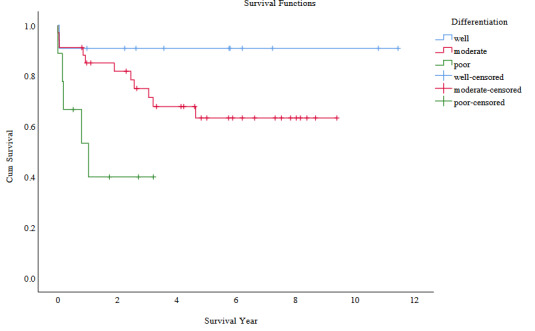
Survival comparison with Kaplan–Meier between the 3 groups of tumor differentiation in beyond the USCF patients (well, moderate and poor).

## 4. Discussion

It is agreed in the literature that one of the most important steps for successful outcomes after LT in HCC is patient selection, as is true in many other areas of medicine [10]. With the improvements in LT, Mazzaferro et al. reported MC for LT in HCC cases in 1996. In this report, survival rates after LT for HCC cases were similar to the survival rates after LT for other diseases [11]. Improved survival rates in patients beyond MC on explant histopathology started the discussion of extending patient selection criteria for LT, as the aforementioned criteria were considered too restrictive. Starting with UCSF [7], many centers began reporting excellent survival rates with their own new criteria [12–25]. LDLT allows many centers to develop center-specific expanded criteria with acceptable results without consideration of allocation system limitations, and LDLT in the setting of HCC has been adopted worldwide over the past decade [9,10,26]. Sugawara et al. utilized a 5-5 rule (up to five nodules with a maximum diameter of 5 cm), and reported a 3-year recurrence-free survival rate of 94% after LDLT [27]. With new limits, Mazzaferro et al. proposed even more liberal criteria than MC: up-to-7 criteria (up to 7 tumors, with the size of the largest tumor up to 7 cm ). They reported that beyond the MC but within up-to-7 criteria in the absence of microvascular invasion had a similar survival rate compared with patients within MC, irrespective of microvascular invasion [28]. Lee et al. reported that beyond the MC with PET-negative status and a total tumor size <10 cm showed similar overall survival and disease-free survival compared to within MC recipients [29]. With the advantage of LDLT, at many centers, especially in Asia, patients with advanced HCC are considered on a case-by-case basis, and risks factors for recurrence, chance of survival, and strong wishes of the patient, donor, and her/his family are considered [30]. However, the selection criteria are still a matter of debate. 

Under the influence of ongoing discussions in the literature, starting with the first case we evaluated, all chronic liver disease patients with HCC were considered case-by-case in our multidisciplinary selection meeting. With the advantage of LDLT, we did not limit our discussions around any criteria. Beyond the tumor size and number, if the patients did not have findings of extrahepatic or macrovascular invasion, tumor thrombosis, lymphatic node or extrahepatic metastasis findings, they were evaluated as an LT candidate. In contrast to DDLT, the indications for LDLT for HCC were decided based on the balance between risks to the living donor and benefits to the recipient [4]. We considered all findings, which provide hints about the biological behavior of the tumor. Tumor growth rate in time, AFP level, tumor margin findings at computed tomography (CT) or magnetic resonance imaging (MRI) views, 18F-labeled fluoro-2-deoxyglucose positron emission tomography (18F-FDG PET) findings, response to other previous treatments, histopathological differentiation (if there was a biopsy) and age of the patients were the parameters we interpreted before making the decision. Only one or two parameters supporting poor biological behavior were not enough to make the decision against LT. The more the morphological limits of selection criteria expand, the more the recurrence rates after LT increase [4]. If most of the findings supported poor biological behavior, alternative and bridge treatment options were suggested instead of LT. In addition, all the possibilities and risks were discussed at length with the recipient, donor candidate and family members. With this evaluation, our survival rates are comparable with the literature and are acceptable. 

According to our analysis, which is also supported widely by the literature, tumor differentiation is the most important factor affecting survival rates. However, biopsy for patients with a decompensated cirrhotic liver is not always possible due to retention of ascites and risk of bleeding as well as tumor dissemination. It could help us to know the tumor differentiation before the decision, but a biopsy cannot accurately diagnose the most advanced degree of differentiation due to the heterogeneity of HCC tumors [4]. Preoperative tumor biopsy and grading analysis have huge variability in specificity and sensitivity, which poses limitations for the prognostic value of biopsy [31]. There is a seeding risk of 3%, false negative rate of 30%, and only 12.5% sensitivity for the identification of microvascular invasion [32,33]. In contrast, the Toronto group reported that the preoperative biopsy is 90% effective in excluding patients with a poorly differentiated lesion. Their recurrence rate related to the preoperative biopsy was 1.9%, which was consistent with previous studies. The Toronto group also reported the biopsy results as one of the main criteria [20]. Dubay et al. reported the usefulness of pretransplant liver biopsy and proposed that LT for advanced moderate to well-differentiated HCC can be performed safely with excellent 5-year overall and disease‑free survival in the absence of size and tumor number restrictions [34]. In our previous short review of our experience correlated to a meeting, we concluded that, considering tumor differentiation, a preoperative biopsy can help select the best HCC patients for transplant even beyond the UCSF criteria with reasonable outcomes [35], but we did not perform routine biopsies in our patients due to the concerns in the literature. Centers’ experiences differ in regard to preoperative tumor biopsy. 

Therefore, noninvasive methods, including tumor markers, CT findings and PET are desirable for predicting the tumor biology. In addition, bridging therapies (transarterial chemoembolization - TACE, transarterial radioembolization -TARE and external beam radiation) prior to LT help control local disease progression [36]. Moreover, imaging modalities have dramatically improved in the last two decades. Some radiologic imaging findings, such as large tumor diameter, tumor margins, the presence of tumor capsule, the distance from tumor to liver capsule, tumor internal homogeneity, contrast enhancement patterns on postcontrast dynamic and hepatobiliary phase images, and diffusion restriction on diffusion weighted images can predict microvascular invasion (MVI). In addition, some clue imaging findings, especially beak and bulging signs, may predict MVI, allowing the clinician to biopsy [37]. We routinely used these noninvasive methods during our evaluation. In some borderline cases, we performed a biopsy for the final decision.

Many earlier studies have shown the importance of vascular invasion as a prognostic marker. Pommergaard HC et al. reported that patients without vascular invasion, regardless of size and number of nodules, had a survival comparable to MC and up-to-7 criteria [32]. On the basis of the idea that incorporating tumor biological markers and predicting microvascular invasion and poor differentiation can exclude patients with a high risk of recurrence before LT, some expanded criteria that include such markers have recently been proposed [20,38,39]. Our data also support these reports in the literature. 

Piardi T. et al reported that tumor size more than 8 cm, AFP level and histologic grading were only independent significant prognostic factors in their LT patients for HCC [31]. With our evaluation system looking at many factors related to poor outcome, we did a limited number of cases with the largest tumor more than 8 cm. In our limited number of cases with the largest tumor size over 8 cm, our data support this literature, with the exception of AFP level. Our experience showed that with the increase in the largest tumor size, other additional poor prognostic factors were seen more often. In addition, when we reviewed our data case by case, an important number of our patients with more than 10 tumors (n = 13) and the largest tumor size greater than 7 cm (n = 11) who underwent LT and were followed long term had the opportunity to live more than 5 years instead of losing their lives much earlier (Table 3 and 4). 

Pre-transplant AFP is independently associated with post‑transplant HCC recurrence survival, suggesting that elevated levels reflect increased tumor aggressiveness that is present even with recurrent disease [40–41]. Elevated AFP is an important prognostic marker associated with the presence of microvascular invasion and poor tumor differentiation [42]. Hong et al. reported that serum AFP levels and ^18^F-FDG PET positivity represent [43], in place of morphological factors, new biological criteria that can improve the risk stratification of tumor recurrence more than the MC for LDLT recipients with HCC [43–44]. Although AFP is the most widely used tumor marker for HCC, only half of all tumors secrete this protein. Thus, AFP may not be an optimal indicator of risk [2]. According to our data, AFP could not be the only marker associated with the poor outcomes. When we looked case by case at our 16 HCC patients with AFP levels higher than 400 mg/mL, remarkably, 14 of them were still alive years after LT (Table 5).

Many new prognostic biomarkers were studied in the literature to establish the outcomes of HCC patients undergoing LT. The most examined biomarker is the serum AFP level. In addition, an association has been found between increased HCC recurrence and high serum levels of Des-gamma –carboxy prothorombin, E-cadherin, beta-catenin and high HCC expression of GPC-3, but additional research is necessary to establish the prognostic role these biomarkers [45]. 

Most of the findings in literature supported that poor biological behavior is the most important impact factor for the outcome. Tumor differentiation is the well-establihed one, which is also supported widely by the literature findings. According to our analysis, tumor differentiation is the only factor that impacts the outcome, which can be a conflict with some of the literature findings such as AFP level, tumor size, 18F-FDG PET, other bimarkers etc. With our evaluation system, we might had a chance to transplant limited number of patients to analyze some of these factors, which might also impact the outcome. This is one of the limitations in our analysis to make a better conclusion. However, we strongly consider a case-by-case basis evaluation for the LT in HCC cases with a multidisciplinary team.

Some studies have suggested that immunosuppression with the mammalian targets of rapamycin (mTOR) inhibitor, such as everolimus or sirolimus, may reduce the risk of HCC recurrence after LT [46]. We followed most of our cases with low tacrolimus levels and switched tacrolimus to mTOR inhibitors in limited recurrence cases. We always tried to treat the recurrent lesions with surgical or interventional radiological treatment. Our experience is limited with mTOR inhibitors for statistical analysis. 

Although overall outcomes are better after LDLT for treatment of HCC, some previous studies had reported that LDLT HCC recipients had worse recurrence compared to DDLT HCC recipients. This was postulated to be due to the lack of ability to test the tumor biology during the waitlist time, which is shorter for LDLT recipients [21,30,47]. Hypotheses include fast-tracking patients to LT, growth factor and cytokines released during the rapid regeneration of a partial graft, surgery technique (may be no-touch total hepatectomy technique). Since LD grafts are not public resources, it is already accepted in the LT community that the recurrence risk of HCC, survival benefit of the recipient, and wishes of the donor should be considered for LDLT candidate selection [30]. In addition, experience with successful LDLT after intensive multidisciplinary treatment for HCC patients with portal vein tumor thrombus, which has been accepted as a contraindication even in the LDLT setting, has been reported in the literature [48–50].

Our endorsement for LDLT would only make sense if we can provide a safe donation environment with a low complication profile. Many centers from Turkey reported their living liver donation complication rates [51–54]. We previously reported complications and outcomes of our 890 living donor hepatectomy cases [8]. No death is reported in our series. Greater experience and knowledge of LDLT will allow reduced donor morbidity. 

 Both the European Association for Study of the Liver (EASL) and American Association for Study of the Liver Disease (AASLD) recently revised guidelines to continue to recommend MC as the benchmark for selection and argue that there is a lack of uniform consensus and limitations inherent to retrospective analysis [55–56]. Literature and guidelines strongly encourage centers moving away from MC to carefully collect prospective data on outcomes using new criteria for selecting patients [57].

## 5. Conclusion

We know that criteria for any medical treatment is important and is usually mandatory. Our data statistically showed that USCF criteria seems more reasonable according to MC. The literature supports LDLT and allows us to push the limits forward. The question “Are the criteria always right?” is always on the table. According to our experience and with the support of the literature, we can conclude that, with the advantage of LDLT, all HCC patients deserve a case-by-case basis discussion for LT under the scientific literature support. In borderline cases, tumor biopsy might help to make a decision about whether to perform LT.
